# Cultivar specific nitrogen and potassium recommendations optimize yield and quality attributes in sugar beet

**DOI:** 10.1038/s41598-025-10918-x

**Published:** 2025-07-29

**Authors:** Sobhi F. Lamlom, Ahmed M. Abdelghany, Aly A. A. El-Banna, Mohamed. M. El-Manhaly, Amr. M. Elsheikh, Noran. A. M. Bassiony, Islam I. Teiba, Gawhara A. El-Sorady, Honglei Ren, Shakeel Ahmad, Taifeng Zhang, Guojun Feng

**Affiliations:** 1 Work Station of Science and Technique for Post-doctoral in Sugar Beet Institute, Heilongjiang University, 74 Xuefu Road, Harbin, 150000 Heilongjiang China; 2 Heilongjiang Junyi Agricultural Limited Liability Company, Harbin, 150000 China; 3 Plant Production Department, Faculty of Agriculture Saba Basha, Alexandria University, Alexandria, 21531 Egypt; 4 Crop Science Department, Faculty of Agriculture, Damanhour University, Damanhour, 22516 Egypt; 5 Sugar Crops Research Institute, Agricultural Research Center, Giza, Egypt; 6 Botany Department, Faculty of Agriculture, Tanta University, Tanta City, 31527 Egypt; 7 Soybean Research Institute of Heilongjiang Academy of Agriculture Sciences, Harbin, 150086 China; 8 State Key Laboratory for Conservation and Utilization of Subtropical Agro-bioresources, College of Life Science and Technology, Guangxi University, Nanning, 530004 China; 9 College of Advanced Agriculture and Ecological Environment, Heilongjiang University, 74 Xuefu Road, Harbin, 150000 Heilongjiang China

**Keywords:** Sugar beet (*Beta vulgaris*), Cultivar selection, PCA, Clustering heatmap, Multivariate analysis, Physiology, Plant sciences, Environmental sciences

## Abstract

This study aimed to investigate cultivar-specific responses to nitrogen and potassium fertilization in sugar beet (*Beta vulgaris*) to optimize yield, quality attributes, and fertilizer use efficiency. Five sugar beet cultivars (Indira, Carma, Mallak, Melodia, and Shantala) were evaluated under nine fertilization treatments combining three nitrogen and potassium levels (T1 (144 N,0 K); T2 (144 N,60 K); T3 (144 N,120 K); T4 (216 N,0 K); T5 (216 N,60K2);T6 (216 N,120 K); T7 (288 N,0 K); T8 (288 N,60 K); T9 (288 N,120 K)) in a split-plot design over two growing seasons (2020/2021 and 202120/22 ). Significant cultivar × treatment interactions (*p* < 0.001) were observed across all parameters. Root yield ranged from 55.72 t/ha (Indira) to 83.33 t/ha (Shantala under T2 treatment). Sucrose content varied from 18.10% (Indira) to 18.92% (Mallak), with corresponding purity levels of 79.93% and 76.39%. Principal component analysis revealed distinct cultivar-specific nutrient responses: lower nitrogen and potassium levels (T1 and T2) positively impacted quality attributes in Indira and Carma, while higher nitrogen and moderate potassium combinations (T3 and T6) enhanced yield attributes in Mallak and Shantala. Economic analysis showed optimal profitability with T3 treatment for Indira and Melodia (ROIs of 89.5% and 91.6%), T2 for Carma and Mallak (ROIs of 123.4% and 113.1%), and T1 or T6 for Shantala (ROI of 166%). Hierarchical clustering and correlation analysis identified strong positive relationships between root yield and biological yield (*r* = 0.94), recoverable sugar yield (*r* = 0.87), and sugar yield (*r* = 0.82). These findings provide valuable recommendations for cultivar-specific nutrient management strategies that farmers can implement to optimize both production efficiency and economic returns while reducing environmental impacts from excessive fertilization.

**Supplementary Information:**

The online version contains supplementary material available at 10.1038/s41598-025-10918-x.

## Introduction

Sugar beet (*Beta vulgaris* L.) is a crop of significant economic importance, accounting for approximately 30% of global sugar production with an output of 42 million MT annually^1^. Beyond its primary role in sugar industries, sugar beet has emerged as a versatile crop used in biofuel production (bioethanol and biomethane) and animal feed, offering diverse economic opportunities^2^. Compared to sugarcane, sugar beet requires 30–40% less water and fertilizer, making it particularly suitable for cultivation in regions with limited water resources^3^.

Despite the known importance of nitrogen (N) and potassium (K) in plant nutrition, limited studies have explored cultivar-specific responses in sugar beet under combined fertilization strategies. Previous research has largely focused on general fertilization recommendations without considering how different cultivars might respond uniquely to varying nutrient combinations. This knowledge gap hinders the development of precision agriculture approaches that could maximize both yield and quality while optimizing resource use^4^. In Egypt, sugar beet has particular significance, contributing approximately 59% of the country’s 2.3 million metric tons of annual sugar production^4,5^. The national agricultural strategy aims to expand sugar beet cultivation to reduce the 30% gap between domestic production and consumption^5,6^. This expansion requires evidence-based fertilization strategies tailored to specific cultivars to maximize productivity under Egyptian conditions.

Chemical fertilizers, particularly those containing nitrogen, have a crucial impact on the process of protein synthesis, which in turn greatly influences the quality of crops^6^. In general, fertilization has a good effect on sugar beets’ water, protein, fiber, and ash content. Simultaneously, it has an inverse impact on the composition of oils, starch, sugars, enzyme activity, and metabolic energy^7,8^. Proper fertilization is essential for the growth and development of field crops, as it assures a consistent supply of nitrogen (N) and other important nutrients^9,10^. Agricultural practitioners employ nitrogen-based fertilizers to enhance soil fertility and maximize the productivity of cultivated crops. Photosynthesis and plant development play a vital role in the growth and productivity of plants^11^.

Nitrogen is one of the three primary essential elements for crop development, along with phosphorus and potassium^12,13^. It directly impacts canopy growth, chlorophyll concentration, and photosynthetic efficiency^14^. This enables farmers to assess the health of their crops promptly and effortlessly^15^. The presence or absence of an adequate amount of nitrogen in sugar beet directly affects the plant’s phenotype^16^. Therefore, a deficiency of nitrogen in sugar beets can be visually detected. In such cases, the canopy fails to reach its complete development, and growth is halted prematurely^17^. The initial signs of nitrogen deprivation manifest on the oldest leaves, resulting in the loss of their green pigmentation^18^. The yellowing spreads along the leaf vein, while the edges of the leaf retain their green color^19^. Conversely, an excessive amount of nitrogen feeding stimulates the growth of the above-ground portion of the plant, leading to an overabundance of leaves^20^. The ratio of detrimental nitrogenous compounds rises as sugar content decreases, leading to a decline in the technological quality of the raw material^8^. Potassium (K+) is considered one of the most crucial phytonutrients for the growth of several crop species, such as sugar beet^21^. K^+^ is an essential microelement vital in coordinating physiological and biochemical processes to help plants withstand environmental challenges such as salinity^22^.

The combined application of N and K is hypothesized to yield better results than individual applications, but the optimal combinations likely vary by cultivar^23,24^. Current fertilization practices often apply uniform recommendations across different sugar beet cultivars, leading to potential resource waste and suboptimal yields^25^. With increasing pressure on agricultural resources and the need for sustainable intensification, cultivar-specific nutrient management represents an important strategy for improving resource use efficiency^26^. This study aimed to investigate the impact of various N and K fertilization rates on both yield and quality parameters across five sugar beet cultivars to develop cultivar-specific fertilization recommendations. Additionally, we conducted economic analyses to provide practical guidance for farmers seeking to optimize both production and profitability. Our findings contribute to the development of precision nutrient management strategies that can enhance sugar beet productivity while improving resource use efficiency in Egypt and similar agroecological zones.

## Materials and methods

### Field trials

Two field experiments were carried out in the two consecutive winter seasons of 2020/2021 and 202120/22 at a private farm in Mutobes region (31°3’ N and longitude 30°39’ E) in Kafer El-Sheikh Governorate. The field investigations were arranged using a split-plot design in a randomized complete block design (RCBD) with three replications. The main plot was assigned to five sugar beet cultivars (iIndira, Carma, Mallak, Melodia, and Shantala) obtained from the Sugar Crop Research Institute, Agricultural Research Center, Giza, Egypt. These cultivars were selected as they represent the most widely grown varieties in Egypt with different yield potentials and quality characteristics. Nine combination of fertilizer contain (three nitrogen rates (urea; 46% N) [144 kg, 216 kg, and 288 kg of nitrogen per hectare] combined with three K rates [0 kg, 60 kg, 120 kg of potassium oxide per hectare] were randomly assigned to the subplots. The nine combination were (T1 (144 N,0 K); T2 (144 N,60 K); T3 (144 N,120 K); T4 (216 N,0 K); T5 (216 N,60K2);T6 (216 N,120 K); T7 (288 N,0 K); T8 (288 N,60 K); T9 (288 N,120 K)). Each experimental plot consists of five ridges, spaced 0.6 m apart and measuring 6.0 m in length, creating an area of 18.0 m2 (3 m x 6 m) with an interplant distance of around 0.20 m within the ridge. Potassium sulfate (K_2_SO_4_; 48% K_2_O) was applied to the soil twice: during planting and 30 days after planting (DFP). Nitrogen fertilizer in the form of (urea; 46% N) as a side dressing in equal quantities. Half of the treatment was administered 35 days post-thinning, while the other half was applied 70 days after planting, right before the third watering. The size of the basic experimental unit was 10.5 m2, consisting of 5 rows of 3.5 m in length and 60 cm in width (i.e., row spacing).

Soil samples were taken at random from the experimental field area at a depth of 15 and 30 cm from soil surface before soil preparation to measure the chemical and physical soil properties as shown in Table 1. Approximately 300 g of soil for ECe measurement was dried, ground, passed through a 10-mesh screen, and saturated with distilled water for 24 h. The pH values of soil samples were measured in saturated soil-water paste using a Bekman pH meter (model Elico, LI120-UK)^27^. Several milliliters of soil-water paste were extracted through a Whitman No. 1 paper filter in Buchner funnel with a vacuum system. The electrical conductivity (EC 25 ◦C) of the soil-paste extracts was determined using a calibrated, temperature-compensating, digital readout conductivity instrument (model 3200, YSI, Inc., Yellow Springs, OH, USA)^27^.

**Table 1:** Physical and chemical properties of the soil experimental sites of 2020 /21 and 2021 /22 seasons.

	Sand%	Silt%	Clay%	Texture class		pH (1:2.5)	EC(m.mhos/cm)		Organic matter %	Available N ppm	Available P ppm	Available K ppm
	**Physical analysis**					**Chemical analysis**						
**2020 /21**	21.68	25.74	52.58	Clay		7.3	2.18		1.3	17	9.3	275.74
**2021/22**	20.32	23.99	55.69	Clay		7	2		2.1	16	9.2	252.54

	SO4 -	CO3-	HCO3 -	Cl -		Ca++	Mg++		Na+	K+	Available B (ppm)	
	**Soluble anions (meq/L)**					**Soluble cations (meq/L)**						
**2020 /21**	5.06	2	3.43	11.31		2.17	8.74		10.34	0.56	0.19	
**2021/22**	6.87	2.14	3.19	8.6		3.65	7.12		9.29	0.64	0.24	

The meteorological data gathered over two growing seasons showed average minimum air temperatures ranging from 9.2 to 10.9 °C, maximum air temperatures ranging from 22.3 to 23.8 °C, relative humidity between 41.0 and 41.6%, wind speeds between 2.0 and 1.8 m sec–1, and precipitation between 0.51 and 0.85 MJ mm day–1.

Healthy seeds of five sugar beet cultivars were obtained from the Sugar Crops Research Institute, Egyptian Agricultural Research Center. Seeds were planted by hand on October 20^th,^ 2020, and 25th 2021 in the first and second seasons, respectively. After sterilization with 1% (v/v) sodium hypochlorite, 3 sugar beet seeds were sown in each hill 20 cm apart. Thirty days after planting (DAS, 4–6 leaf stage), the seedlings were thinned to one per hill to reach approximately 83,000 plants ha^− 1^. Harvesting was done by gathering roots on April 25^th,^ 2021, and 28th 2022. Thinning was conducted at the 4-leaf stage (about 30 days after planting) to maintain one plant per hill. Plants underwent irrigation using a surface irrigation system during their growth and development. The recommended practices, disease control, fertilization program, and pest management were the same as those used in local commercial sugar beet cultivation.

### Sampling and measuring yield and quality traits

Sampling was done in the two seasons at 210 DAS (Standard harvest timing) from all sub plots in both seasons to evaluate the yield and quality traits. Each sample consists of six randomly selected plants and completely removed after irrigation of the soil to facilitate obtaining the plant with the whole root. Sugar beet plants from all rows were then collected in each sub-sub plot, plus the six previously sampled plants that were all used to measure yield traits.

#### Yield traits

Sugar beet plants from all rows of each sub-sub-plot were weighed, in addition to weighing 6 plants that were previously sampled and then converted to root yield (RY, t/ha), along with biological yield (BY, t/ha), which was computed by adding the root yield to the top yield (t/ha). Total sugar yield (SY, t/ha) was calculated by multiplying the RY by the total sugar (%).

#### Quality traits

Samples of the six sugar beet roots were taken randomly from each sub-plot, washed, and dried to calculate the following parameters: The sucrose content (SC %) according to^28^. Sugar loss to molasses (SLM %), purity (%), and alkalinity index (AC) were calculated by the following equations:

-SLM (%) = gross sugar (%) - pure sugar (%).
*-Purity (%) = [Pure sugar (%) ÷ Gross sugar (%)] × 100*.
*-AC= (K + Na) ÷ α-amino N accordingt to*^29^.

The content of impurities in terms sodium (Na), K, and α-amino-N in (meq per 100 g root) were determined by an Automatic Sugar Polarimetric. The recoverable sugar yield (RSY, t/ ha) was calculated using the following equation : Recoverable sugar yield = RY x sugar recovery.

where sugar recovery % (SR %) was calculated using by subtracting sucrose content % - sugar loss % according^30^. Sugar analysis typically employs two key instruments: the automatic sugar polarimetric analyzer, which measures optical rotation to determine sucrose purity (reported in degrees Z or % pol) following ICUMSA Method GS1/2/3 − 1 protocols, and the hand refractometer, which measures Total Soluble Solids (TSS) through refractive index (reported as % Brix) following ICUMSA Method GS4/3–13 standards. Together, these instruments enable comprehensive sugar quality assessment through critical parameters including pol (sucrose content), Brix (total soluble solids), purity ((Pol ÷ Brix)×100%), reducing sugars (g/100 g), pH (dimensionless, typically 5.0–7.0), color (ICUMSA Units), moisture content (%), and ash content (%), with measurements validated through standardized ICUMSA and AOAC methodologies that ensure consistent quality control across the sugar industry.

### Economic analysis

**Input Costs**.

We calculated the costs of fertilizer inputs based on current market prices: Urea fertilizer (46% N): $450 per ton and Potassium sulfate (48% K₂O): $650 per ton.

Additional costs considered:

Application costs: $15 per hectare per application.

Labor costs: $25 per labor-day.

Other cultivation costs (seed, irrigation, pest management, etc.): $1,200 per hectare (constant across treatments).

Total fertilizer costs were calculated for each treatment:

T1 (N1K1): $396 per hectare.

T2 (N1K2): $435 per hectare.

T3 (N1K3): $474 per hectare.

T4 (N2K1): $498 per hectare.

T5 (N2K2): $537 per hectare.

T6 (N2K3): $576 per hectare.

T7 (N3K1): $600 per hectare.

T8 (N3K2): $639 per hectare.

T9 (N3K3): $678 per hectare.

**Revenue Calculation**.

Revenue was calculated based on:

Root yield (t/ha).

Base price for sugar beet: $45 per ton.

Quality premium/penalty: ±$0.80 per percentage point of sucrose content above/below 16%.

Processing efficiency bonus: +$1.20 per percentage point of purity above 75%.

The revenue formula used:

Revenue = Root Yield × [Base Price + (Sucrose Content − 16) × 0.8 + (Purity − 75) × 1.2]

**Profitability Metrics**.

For each cultivar-treatment combination, we calculated:

Gross margin ($/ha) = Revenue - Total input costs.

Benefit-cost ratio (BCR) = Revenue ÷ Total input costs.

Return on investment (ROI, %) = (Gross margin ÷ Total input costs) × 100.

Marginal return ($/kg) = Change in gross margin ÷ Change in fertilizer amount.

### Statistical analysis

Analysis of variance (ANOVA) at a significant level of *P* < 0.05 was performed with SAS 9.4 (SAS Institute Inc., Cary, NC, United States) to statistically analyze the data obtained following the split-split plot arranged in RCBD. Tukey’s test was also applied in SAS to compare treatments which means at probability of *P* < 0.05, and further presented in boxplots and barplots which were constructed using *ggplot2* package in software R (R Core Team, version 4.1.1, 2021). A two-way hierarchical clustering heatmap was conducted using the R package Complex Heatmap. In addition, the R package *corrplot* was implemented to analyze correlation matrix plot, while the two R packages FactoMineR and factoextra were used to generate principal component analysis (PCA) biplot.

## Results

### Variation in yield and quality traits among sugar beet cultivars

**Fig. 1:**
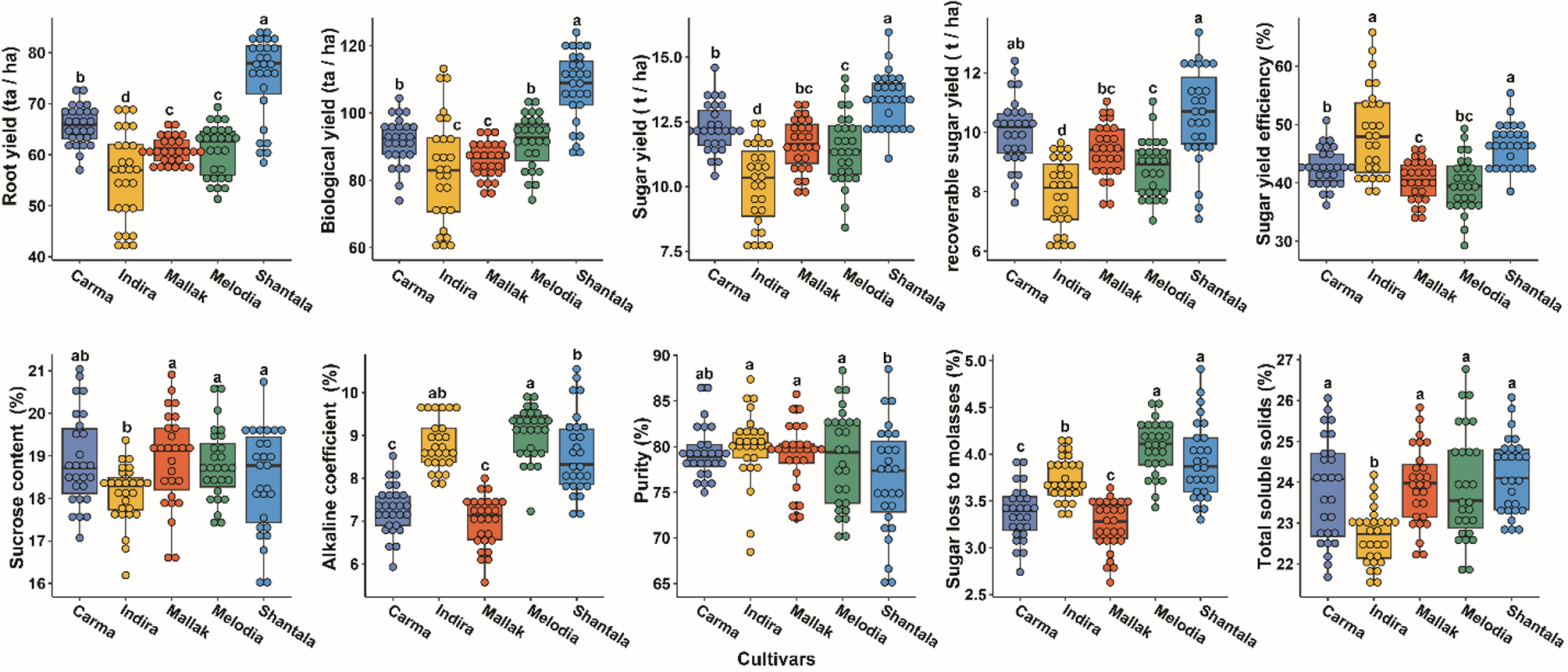
Variation in Yield and Quality Traits Among Sugar Beet Cultivars. Boxplots depicting the distribution of root yield (RY, t/ha), biological yield (BY, t/ha), (C) sucrose content (SC, %), alkaline coefficient (AC), purity (%), shoot-to-root ratio (SLM), recoverable sucrose yield (RSY, t/ha), sugar yield (SY, t/ha), and total soluble solids (TSS, %) across five sugar beet cultivars (Indira, Carma, Mallak, Melodia, and Shantala). The boxplots show the median (horizontal line), interquartile range (box), and minimum/maximum values (whiskers), with outliers represented as individual points.

The performance of five sugar beet cultivars (Indira, Carma, Mallak, Melodia, and Shantala) was evaluated across ten yield and quality traits (Fig. 1). Significant variations were observed among the cultivars for these crucial agronomic traits. Root yield (RY), ranged from 55.72 t/ha in Indira to 75.16 t/ha in Shantala. Cultivars Carma (65.80 t/ha), Mallak (60.92 t/ha), and Melodia (60.69 t/ha) exhibited moderate root yields. A similar trend was observed for biological yield (BY), with Shantala (107.76 t/ha) displaying the highest biomass accumulation, followed by Carma (91.48 t/ha) and Melodia (91.59 t/ha). Regarding sugar yield (SY), Shantala outperformed the other cultivars, with a yield of 13.35 t/ha. Carma (12.27 t/ha) and Mallak (11.63 t/ha) exhibited relatively high sugar yields, while Indira (10.07 t/ha) recorded the lowest level. The recoverable sugar yield (RSY) followed a similar pattern, with Shantala (10.58 t/ha), whereas Indira (7.93 t/ha) had the lowest RSY value. For sugar yield efficiency (SYE), the levels ranged from 39.73% in Melodia to 48.48% in Indira to 48.48% in Indira. Regarding sugar quality traits, Mallak and Indira displayed the highest sucrose content (SC) (18.92%) and purity level (79.93%), while Indira (18.10%) and Shantala (76.39%) scored the lowest levels of both quality parameters. The alkaline coefficient (AC), an indicator of impurities, was lowest in Carma (6.99%) and highest in Melodia (9.05%). Sugar loss to molasses (SLM), another measure of processing efficiency, ranged from 3.24% in Mallak to 4.07% in Melodia. For total soluble solids (TSS) content was highest in Shantala (24.17%) and lowest in Indira (22.67%), with the remaining cultivars exhibiting intermediate levels. In summary, Shantala demonstrated superior performance in terms of root yield, biological yield, and sugar yield, making it a promising cultivar for high productivity. However, Mallak and Indira showed better sugar quality traits, such as sucrose content and purity, which are critical for processing efficiency. These findings highlight the importance of selecting cultivars based on specific yield and quality objectives in sugar beet production.

### Impact of nitrogen and potassium fertilization on sugar beet yield and quality traits

**Fig. 2:**
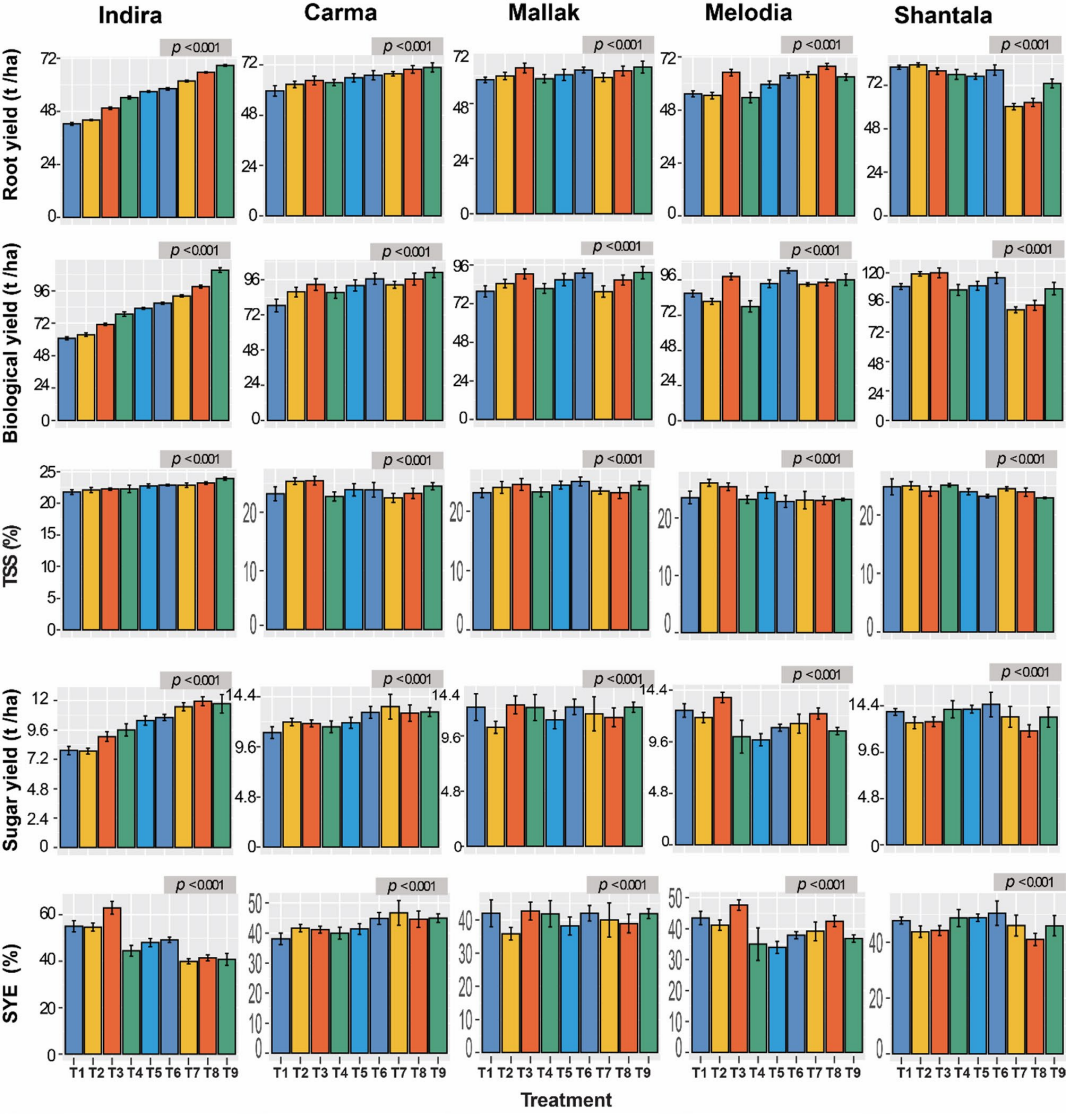
Effect of Nitrogen and Potassium Fertilization on Yield and Quality Traits of Five Sugar beet Cultivars. RY : root yield (t / ha), BY : biological yield (t / ha), TSS : total soluble solids (%), SY: sugar yield (t / ha), SYE: sugar yield efficiency (%). T1: N1K1, T2: N1K2, T3: N1K3, T4: N2K1, T5: N2K2, T6: N2K3, T7: N3K1, T8: N3K2, T9: N3K3. Statistically significant differences (*p* < 0.001) were observed between treatments for all cultivars.

**Fig. 3:**
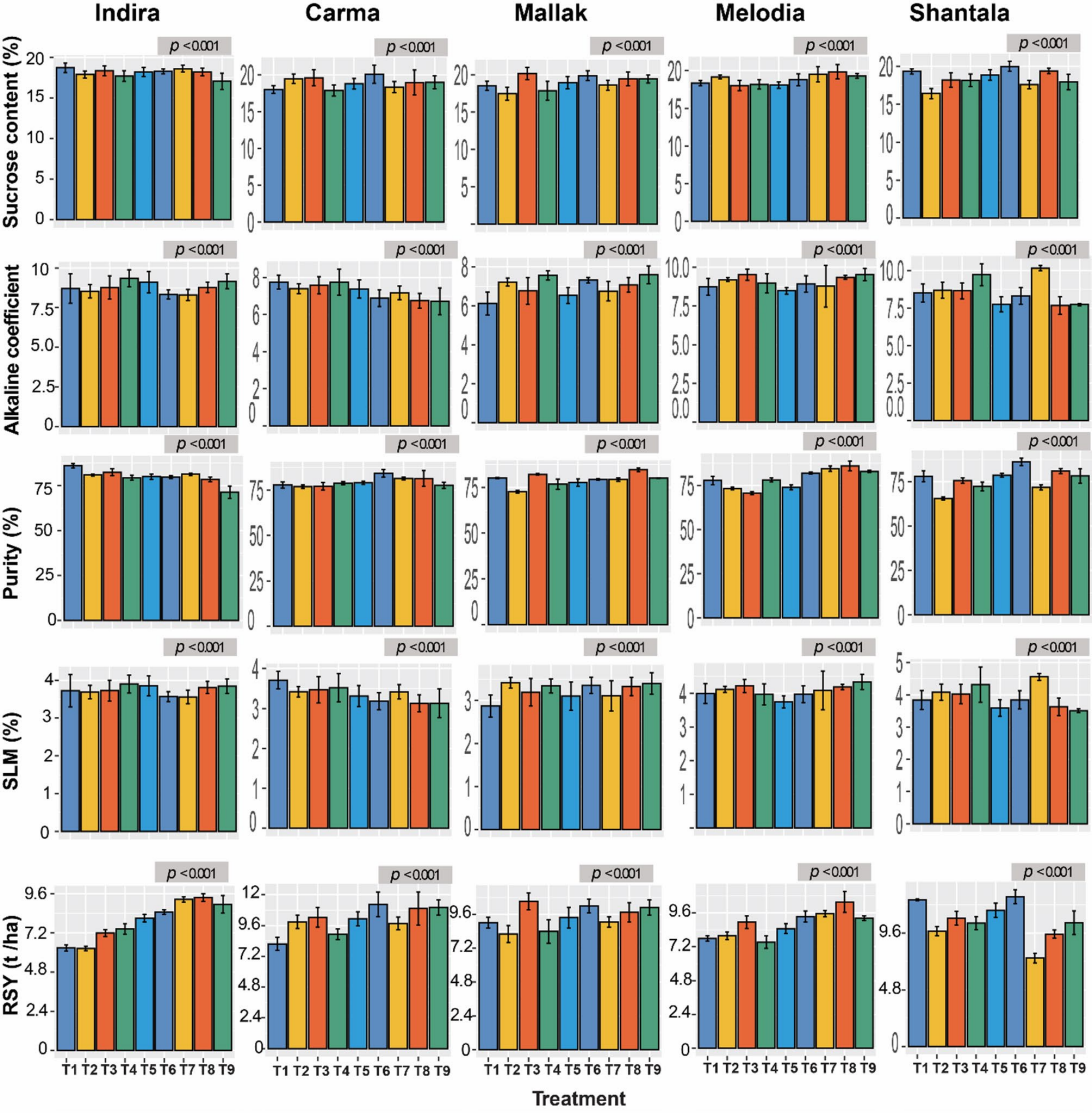
Effect of Nitrogen and Potassium Fertilization on Yield and Quality Traits of Five Sugar beet Cultivars. AC : alkaline coefficient, SLM : Loss of sugar to molasses (%), RSY: Recoverable sugar (ton / ha). T1: N1K1, T2: N1K2, T3: N1K3, T4: N2K1, T5: N2K2, T6: N2K3, T7: N3K1, T8: N3K2, T9: N3K3. Statistically significant differences (*p* < 0.001) were observed between treatments for all cultivars.

The application of varying levels of nitrogen (N) and potassium (K) fertilizers significantly influenced the yield and quality traits of the five sugar beet cultivars, as shown in Fig. 2. The findings showed significant variations (*p* < 0. 001) observed in all the characteristics across all the cultivar x fertilizer treatment combinations that were examined. It can be ascertained that the values of N and K, which are pertinent to the specific cultivar as well as the goals, are likely to differ. Accordingly, it was observed that Shantala yielded the highest RY with treatment combination T2 (83.33 t/ha), followed by treatment combination T1 (81.96 t/ha), in comparison to all other fertilization treatments. Trends in BY were also closely similar to that found with RY, as the two treatments of T2 (119.17 t/ha) and T3 (119.96 t/ha) showed the highest BY for Shantala cultivar. TSS was in its maximum when the cultivar Melodia was treated with T2, achieving a level of 26.17%. The treatment combinations also differed noticeably in respect to SYE as the cultivar Indira treated with T3 recorded 62.94%, showing the highest level of such parameter. The highest level of SY was obtained by T6, as it recorded 14.55 t/ha in the cultivar Shantala. Figure 2 also illustrates the remaining traits, including SC, purity, AC, SLM, and RSY. For SC, Mallak cultivar exhibited the highest average SC (S) across all treatments, with a maximum value exceeding 20.14 at treatment T3 (Fig. 3). Regarding AC,. The trends in alkaline coefficient (AC) across cultivars and treatments were not entirely consistent. While some cultivars like Indira showed a decreasing trend in AC with increasing N and K levels (T1 to T3), others like Melodia exhibited the opposite trend (Fig. 3). The treatment T1 when applied to the cultivar Mallak recorded the lowest AC level of 6.12%, while the highest AC was recorded by Shantala when treated with T7 (10.15%). Furthermore, the cultivar Shantala treated with T6 achieved the highest sugar beet purity (86.16%). For SLM, similarly to AC trait, the cultivar Mallak treated with T1 treatment recorded the lowest SLM (2.87%). Finally, Shantala cultivar also achieved the highest level of RSY (12.68 t/ha) when applied with the same treatment combination (T6). In summary, the application of N and K fertilizers significantly influenced the yield and quality traits of sugar beet cultivars, with Shantala demonstrating superior performance under specific treatment combinations (T2 and T6). However, cultivar-specific responses were evident, highlighting the importance of tailored fertilization strategies to optimize yield and quality. These findings provide valuable insights for precision nutrient management in sugar beet production.

### Principal component analysis reveals Cultivar-Specific responses to nitrogen and potassium fertilization levels

**Fig. 4:**
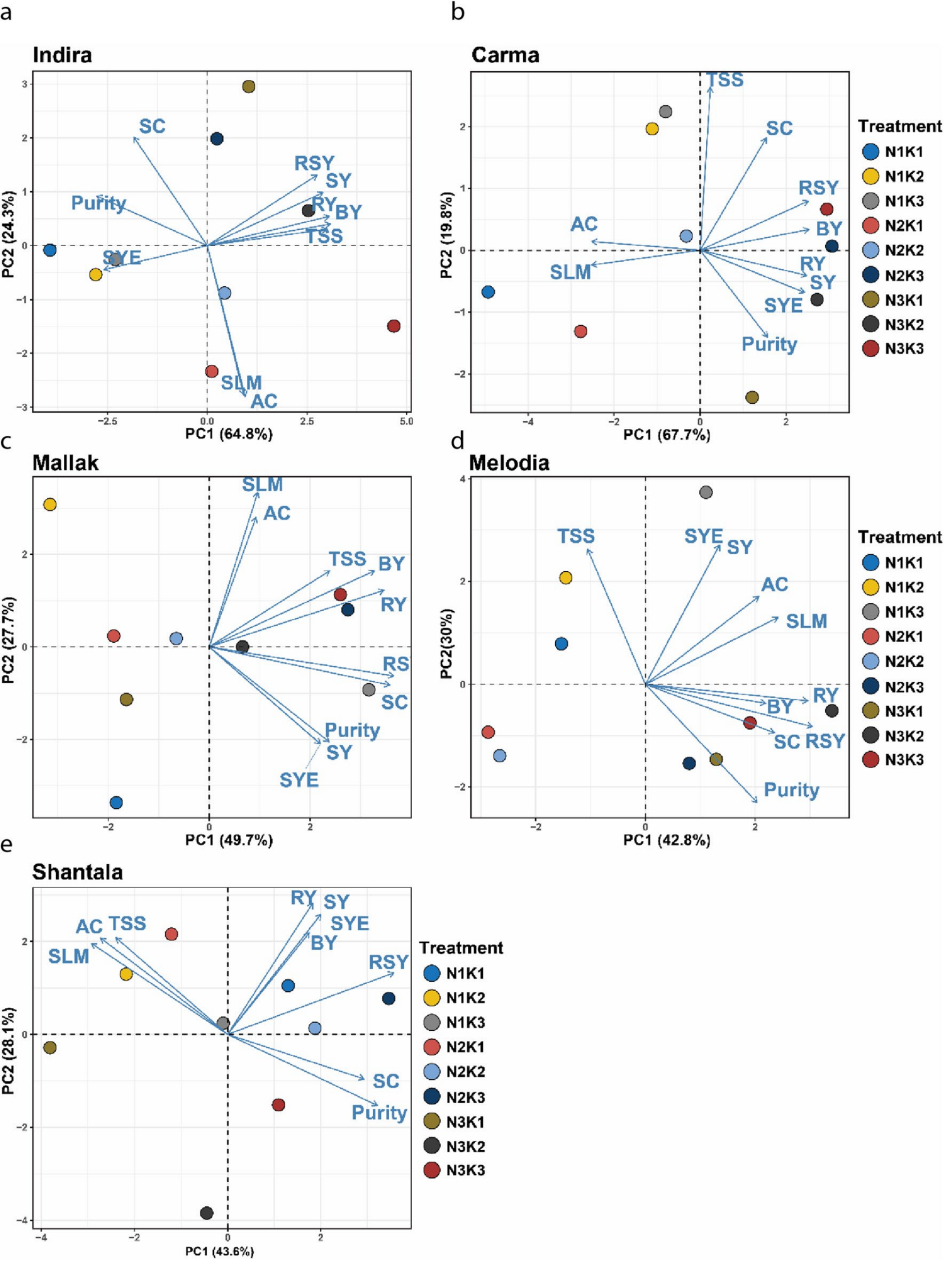
Principal component analysis biplots depict cultivar-specific relationships among yield, quality traits, and nitrogen-potassium fertilization regimes in five sugar beet cultivars (Indira, Carma, Mallak, Melodia, and Shantala). The first principal component (PC1) and its corresponding percentage of explained variance are shown on the x-axis for each cultivar. Traits like root yield (RY), biological yield (BY), total soluble solids (TSS), sugar yield (SY), sugar yield efficiency (SYE), sucrose (S), alkaline coefficient (AC), purity, sugar loss to molasses (SLM), and recoverable sugar yield (RSY) are represented by labeled points. The nine nitrogen-potassium treatment combinations (N1K1, N1K2, N1K3, N2K1, N2K2, N2K3, N3K1, N3K2, and N3K3) are color-coded for visual distinction.

The principal component analysis (PCA) revealed intricate patterns and relationships among yield, quality traits, and nutrient treatment combinations, highlighting cultivar-specific responses in each of the five sugar beet cultivars (Fig. 4a-e). For the cultivar Indira, the first principal component (PC1) accounted for 64.8% of the total variance and distinctly separated SC, purity, RSY, and SY from SLM and AC. Distinctly, BY and TSS occupied an intermediate position along PC1. This pattern suggests that for Indira, increasing sucrose, purity, RSY, and SY could potentially come at the expense of higher SLM and AC values (Fig. 4a). In the case of Carma (Fig. 4b), PC1 (67.7% of variance) contrasted sucrose, RSY, and BY against SLM, purity, and SYE, with AC loadings near zero. The AC trait appeared less influential in driving the variation along PC1 for Carma. For Mallak (Fig. 4c), PC1 (49.7%) differentiated the undesirable traits SLM and AC from TSS, BY, RY, sucrose, purity, SY, and SYE. This pattern suggests that in Mallak, efforts to improve TSS, BY, RY, S, purity, SY, and SYE could potentially lead to increased SLM and AC levels. The cultivar Melodia exhibited a similar pattern, with PC1 (42.8%) separating the desirable traits SYE, SY, and SLM from RY, BY, S, RSY, and purity (Fig. 4d). Interestingly, the cultivar Shantala displayed a distinct pattern along PC1 (43.6%), contrasting the traits SLM, AC, and TSS against the favorable attributes RY, SY, SYE, BY, and RSY (Fig. 4e). This suggests that efforts to enhance RY, SY, SYE, BY, and RSY in Shantala could potentially lead to higher SLM, AC, and TSS levels. For illustrating the magnitude of each treatment combination of N and K with their different levels, the biplots also highlighted cultivar-specific responses to the nutrient treatment combinations. For instance, in Indira and Carma, the N1K1 and N1K2 treatments grouped closely with favorable traits like S and RSY, while N3K3 associated with undesirable traits like SLM and AC. This implies that lower nitrogen and potassium levels (N1K1 and N1K2) may be more beneficial for these cultivars in terms of maximizing S and RSY while minimizing SLM and AC. Conversely, in Mallak and Shantala, the N3K2 and N3K3 treatments aligned more closely with desirable yield and quality attributes like RY, SY, and SYE. This suggests that higher nitrogen and moderate to high potassium levels (N3K2 and N3K3) could be more advantageous for these cultivars in optimizing yield and quality parameters. The cultivar Melodia exhibited a slightly different pattern, with the N1K3 treatment grouping closely with favorable traits like SYE, SY, and SLM, while N3K1 is associated with purity. In summary, PCA revealed distinct cultivar-specific responses to nitrogen and potassium treatments, emphasizing the importance of tailored nutrient management strategies. Lower N and K levels (N1K1 and N1K2) were beneficial for Indira and Carma in enhancing sucrose and RSY, while higher N and K levels (N3K2 and N3K3) optimized yield and quality traits in Mallak and Shantala. These findings underscore the need for cultivar-specific fertilization approaches to achieve optimal yield and quality in sugar beet production.

### Clustering heatmap analysis of nitrogen and potassium effects on sugar beet traits

**Fig. 5:**
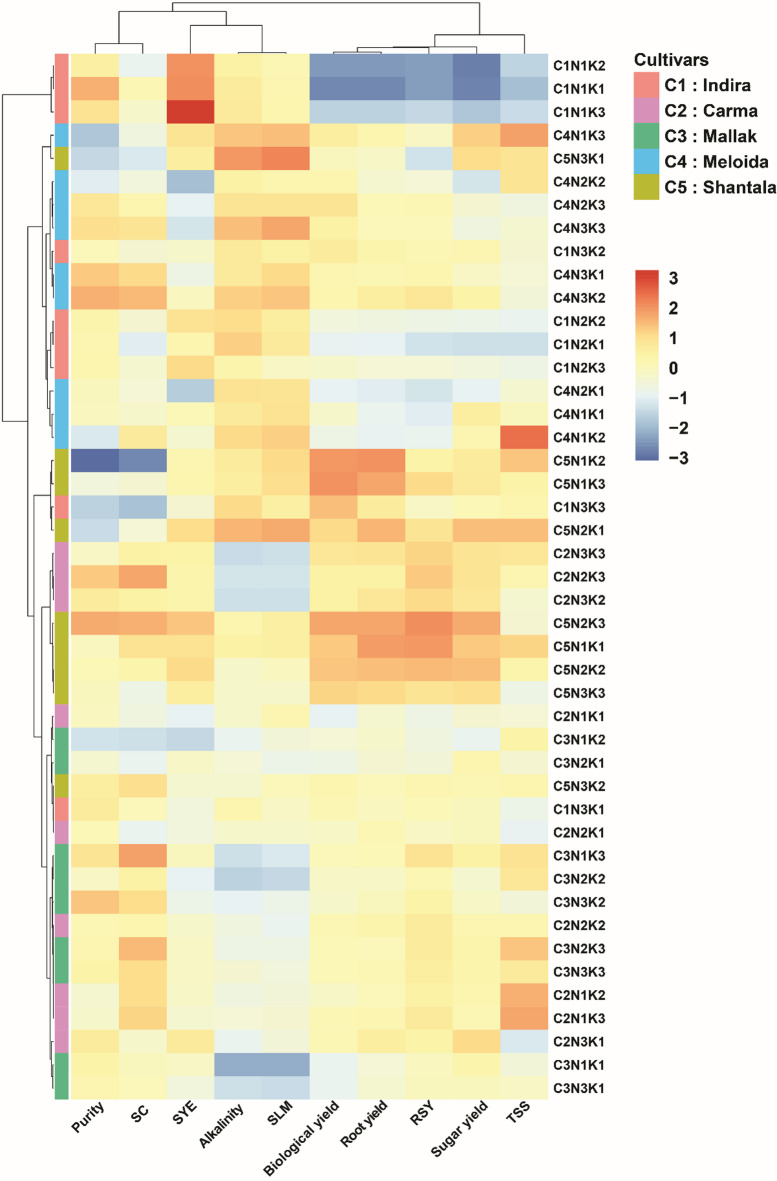
Hierarchical clustering heatmap illustrating the interrelationships between nitrogen-potassium fertilization treatment combinations (N1K1, N1K2, N1K3, N2K1, N2K2, N2K3, N3K1, N3K2, and N3K3) and yield and quality traits across five sugar beet cultivars (C1: Indira, C2: Carma, C3: Mallak, C4: Melodia, and C5: Shantala). The heatmap depicts the clustering patterns of the treatment combinations based on their associations with traits such as root yield (RY), biological yield (BY), total soluble solids (TSS), sugar yield (SY), sugar yield efficiency (SYE), SC (S), alkaline coefficient (AC), purity, sugar loss to molasses (SLM), and recoverable sugar yield (RSY). The color scale represents the strength of the associations, with red indicating positive relationships and blue representing negative relationships. The dendrogram on the left side illustrates the hierarchical clustering of the treatment combinations based on their similarity in trait associations across cultivars.

The hierarchical clustering (Fig. 5) analysis provided insights into the interrelationships between the nitrogen-potassium treatment combinations (N1K1, N1K2, N1K3, N2K1, N2K2, N2K3, N3K1, N3K2, and N3K3) and the studied yield and quality traits across the five sugar beet cultivars (C1: Indira, C2: Carma, C3: Mallak, C4: Melodia, and C5: Shantala). Root and biological yield formed a distinct cluster separate from the other traits. This tight grouping indicates these two yield parameters responded similarly across all cultivars regardless of fertilizer levels, with close correlation between the variables. Another major cluster was evident towards the middle of the heatmap, comprised of TSS, SY, S, and RSY, signifying moderate to strong positive relationships between these traits. As expected, TSS, SY, and S are logically associated as indicators of sugar content and productivity in sugar beets. Interestingly, RSY is also correlated well with this group rather than clustering independently as might have been predicted. Moving higher up the heatmap, AC and SLM formed an interlinked cluster, pertaining to sugar processing capabilities thus co-varied significantly across the fertilizer-cultivar matrix. Purity tended to behave independently from the other traits based on its isolation in a cluster of light purple cells in the upper left corner. This divergent response suggests loss to molasses is regulated by distinct underlying mechanisms compared to the other evaluated characteristics. At the level of cultivar and fertilizations combinations, the treatment combinations N1K1, N1K2, and C1N1K3 for cultivar C1 (Indira) formed a distinct cluster, exhibiting strong positive associations with desirable traits such a S, purity, SY, RSY, RY. In contrast, the N3K3 treatment grouped separately, aligning more closely with undesirable traits like SLM and AC. The cultivar C2 (Carma) displayed a similar pattern, with N1K1 and N1K2 clustering together and exhibiting positive relationships with favorable traits like S, RSY, and BY. Conversely, the N3K2, N2K3, and N3K3 treatment showed negative association with unfavorable traits such as AC and SLM. In the case of cultivar C3 (Mallak), the N3K2 and N3K3 treatments formed a distinct cluster, showing positive associations with desirable traits like TSS, BY, RY, and S. However, these treatments also exhibited some affinity towards undesirable traits like SLM and AC. For cultivar C4 (Melodia), the N3K1 treatment emerged as a distinct group, aligning favorably with most of the studied traits except for TSS and SYE. Conversely, the N3K1 treatment showed closer relationships with all desirable and undesirable traits, except for S. The cultivar C5 (Shantala) exhibited a unique pattern, with the N1K3 treatments displayed positive associations with favorable traits like RY, SY, SYE, BY, and RSY, while N1K2 aligned positively with more favorable traits, however showed negative association with both purity and SC. In summary, hierarchical clustering revealed distinct patterns in the relationships between yield, quality traits, and nutrient treatments across the five sugar beet cultivars. Lower nitrogen and potassium levels (N1K1, N1K2, and N1K3) were associated with favorable traits in Indira and Carma, while higher nitrogen and potassium levels (N3K2 and N3K3) optimized yield and quality in Mallak and Shantala. These findings highlight the importance of cultivar-specific nutrient management strategies to maximize desirable traits and minimize undesirable ones in sugar beet production. In summary, hierarchical clustering revealed distinct patterns in the relationships between yield, quality traits, and nutrient treatments across the five sugar beet cultivars. Lower nitrogen and potassium levels (N1K1, N1K2, and N1K3) were associated with favorable traits in Indira and Carma, while higher nitrogen and potassium levels (N3K2 and N3K3) optimized yield and quality in Mallak and Shantala. These findings highlight the importance of cultivar-specific nutrient management strategies to maximize desirable traits and minimize undesirable ones in sugar beet production.

### Correlation analysis unveils intricate relationships among yield and quality traits in sugar beet

**Fig. 6:**
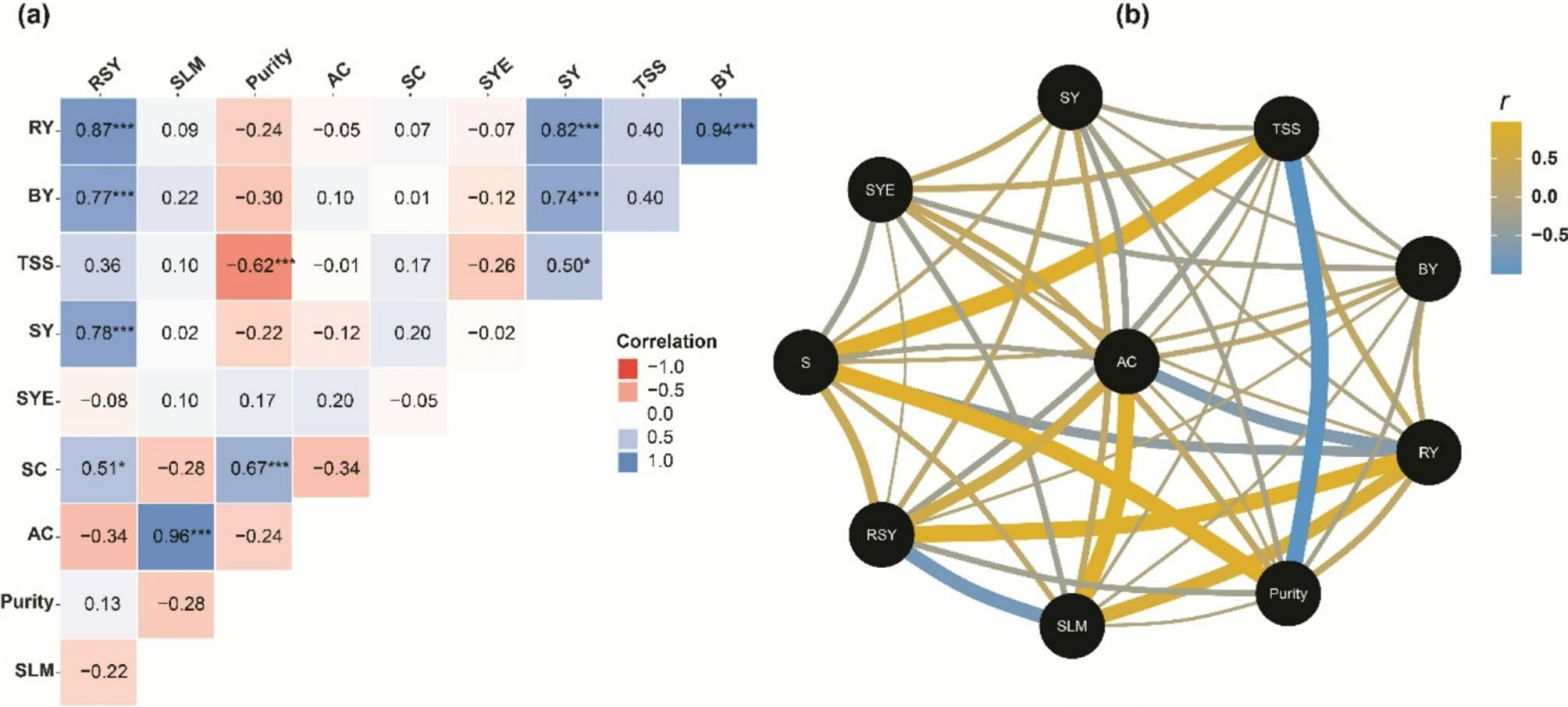
Correlation matrix (**a**) and network visualization (**b**) provide illustrating the relationships among key yield and quality traits in sugar beets, including root yield (RY), biological yield (BY), total soluble solids (TSS), sugar yield (SY), sugar yield efficiency (SYE), SC, alkaline coefficient (AC), purity, sugar loss to molasses (SLM), and recoverable sugar yield (RSY). In the correlation matrix (a) the correlation coefficients are color-coded, with shades of blue indicating positive correlations and shades of red representing negative correlations. The intensity of the colors corresponds to the strength of the correlation, with darker shades denoting stronger relationships. Asterisks (***, **, *) indicate statistical significance at *p* < 0.001, *p* < 0.01, and *p* < 0.05, respectively.

The results presented in Fig. 6 display a correlation matrix (Fig. 6a) and correlation network (Fig. 6b), showcasing the relationships between various yield and quality traits in sugar beets. The correlation analysis revealed significant relationships among various yield and quality traits in sugar beet. RY exhibited a strong positive correlation with BY (*r* = 0.94***), RSY (*r* = 0.87***), and SY (*r* = 0.82***), indicating that higher root biomass production was associated with increased sugar accumulation and overall yield. In addition, TSS showed a moderate positive correlation with SY (*r* = 0.50*) but was negatively correlated with SLM (*r* = -0.62***), suggesting that higher TSS levels could contribute to enhanced sugar yield while reducing sugar loss during processing. SC had a strong positive correlation with purity (*r* = 0.67***) but a negative correlation with SLM (*r* = -0.28) and AC (*r* = -0.34). This implies that higher sucrose levels are desirable for improving purity and reducing sugar loss and impurity levels in sugar beets. Interestingly, AC exhibited a strong positive correlation with purity (*r* = 0.96***), indicating that higher AC values could be associated with increased purity, despite the negative correlation between AC and SC. This relationship warrants further investigation to understand the underlying mechanisms. Also, SYE showed a moderate positive correlation with AC (*r* = 0.20) and purity (*r* = 0.17), suggesting that optimizing these parameters could potentially enhance the efficiency of sugar extraction and recovery. The correlation analysis highlighted strong interdependencies among yield and quality traits in sugar beets. Root yield (RY) and biological yield (BY) were closely linked to sugar accumulation, while sucrose content (SC) and purity were critical for improving processing efficiency and reducing sugar loss. The unexpected positive correlation between AC and purity requires further exploration to elucidate its implications for sugar beet quality. These findings provide valuable insights for optimizing yield and quality traits through targeted breeding and agronomic practices.

### Economic performance by cultivar and treatment

**Table 2:** Economic performance of fertilization treatments by Cultivar.

Cultivar	Treatment	Root Yield (t/ha)	Sucrose (%)	Purity (%)	Input Cost ($/ha)	Revenue ($/ha)	Gross Margin ($/ha)	BCR	ROI (%)
Indira	T1	52.63	18.45	81.36	1,596	2,895	1,299	1.81	81.4
	T2	54.82	18.32	80.21	1,635	2,962	1,327	1.81	81.2
	T3	58.70	17.49	82.94	1,674	3,173	1,499	1.90	89.5
	T4	53.16	17.94	78.63	1,698	2,789	1,091	1.64	64.3
	T5	54.97	17.81	77.45	1,737	2,840	1,103	1.64	63.5
	T6	57.13	16.85	76.28	1,776	2,823	1,047	1.59	59.0
	T7	53.69	17.42	75.94	1,800	2,730	930	1.52	51.7
	T8	57.84	17.31	75.16	1,839	2,897	1,058	1.58	57.5
	T9	59.12	16.36	74.37	1,878	2,776	898	1.48	47.8
Carma	T1	64.27	18.91	79.85	1,596	3,611	2,015	2.26	126.3
	T2	66.14	18.74	78.96	1,635	3,653	2,018	2.23	123.4
	T3	67.85	18.12	77.23	1,674	3,625	1,951	2.17	116.5
	T4	62.68	18.37	76.52	1,698	3,349	1,651	1.97	97.2
	T5	65.30	18.21	75.74	1,737	3,449	1,712	1.99	98.6
	T6	67.86	17.69	75.13	1,776	3,515	1,739	1.98	97.9
	T7	63.45	17.82	74.52	1,800	3,237	1,437	1.80	79.8
	T8	66.91	17.64	73.92	1,839	3,345	1,506	1.82	81.9
	T9	68.73	17.23	73.35	1,878	3,358	1,480	1.79	78.8
Mallak	T1	56.32	20.14	83.27	1,596	3,341	1,745	2.09	109.3
	T2	59.85	19.87	82.13	1,635	3,484	1,849	2.13	113.1
	T3	61.27	19.52	80.94	1,674	3,498	1,824	2.09	109.0
	T4	58.43	19.36	79.81	1,698	3,278	1,580	1.93	93.1
	T5	60.19	19.14	78.74	1,737	3,327	1,590	1.92	91.5
	T6	62.78	18.75	77.65	1,776	3,402	1,626	1.92	91.6
	T7	59.24	18.96	76.92	1,800	3,223	1,423	1.79	79.1
	T8	62.35	18.68	76.12	1,839	3,330	1,491	1.81	81.1
	T9	64.14	18.32	75.35	1,878	3,370	1,492	1.79	79.4
Melodia	T1	57.25	18.23	78.64	1,596	3,054	1,458	1.91	91.4
	T2	59.46	18.15	77.86	1,635	3,126	1,491	1.91	91.2
	T3	61.84	17.98	77.12	1,674	3,207	1,533	1.92	91.6
	T4	58.13	17.76	76.45	1,698	2,975	1,277	1.75	75.2
	T5	60.72	17.54	75.84	1,737	3,041	1,304	1.75	75.1
	T6	62.97	17.39	75.26	1,776	3,116	1,340	1.75	75.5
	T7	59.34	17.32	74.86	1,800	2,913	1,113	1.62	61.8
	T8	61.98	17.16	74.35	1,839	2,989	1,150	1.63	62.5
	T9	64.51	16.89	73.92	1,878	3,054	1,176	1.63	62.6
Shantala	T1	81.96	17.41	79.52	1,596	4,246	2,650	2.66	166.0
	T2	83.33	17.32	78.95	1,635	4,276	2,641	2.62	161.5
	T3	77.95	17.18	78.43	1,674	3,968	2,294	2.37	137.0
	T4	70.63	17.09	77.96	1,698	3,574	1,876	2.10	110.5
	T5	72.46	16.92	77.42	1,737	3,622	1,885	2.09	108.5
	T6	79.58	16.78	86.16	1,776	4,290	2,514	2.42	141.6
	T7	70.84	16.54	76.37	1,800	3,462	1,662	1.92	92.3
	T8	73.16	16.38	75.86	1,839	3,529	1,690	1.92	91.9
	T9	75.52	16.17	75.31	1,878	3,608	1,730	1.92	92.1

### Marginal returns analysis

The marginal return analysis evaluated the economic benefit of additional fertilizer input across treatments:

**Table 3:** Marginal returns for nitrogen and potassium Application.

Cultivar	Comparison	ΔN (kg/ha)	ΔK (kg/ha)	ΔYield (t/ha)	ΔGross Margin ($/ha)	Marginal Return ($/kg)
Indira	T1 → T2	0	60	2.19	28	0.47
	T2 → T3	0	60	3.88	172	2.87
	T1 → T4	72	0	0.53	-208	-2.89
	T4 → T7	72	0	0.53	-161	-2.24
Carma	T1 → T2	0	60	1.87	3	0.05
	T2 → T3	0	60	1.71	-67	-1.12
	T1 → T4	72	0	-1.59	-364	-5.06
	T4 → T7	72	0	0.77	-214	-2.97
Mallak	T1 → T2	0	60	3.53	104	1.73
	T2 → T3	0	60	1.42	-25	-0.42
	T1 → T4	72	0	2.11	-165	-2.29
	T4 → T7	72	0	0.81	-157	-2.18
Melodia	T1 → T2	0	60	2.21	33	0.55
	T2 → T3	0	60	2.38	42	0.70
	T1 → T4	72	0	0.88	-181	-2.51
	T4 → T7	72	0	1.21	-164	-2.28
Shantala	T1 → T2	0	60	1.37	-9	-0.15
	T2 → T3	0	60	-5.38	-347	-5.78
	T1 → T4	72	0	-11.33	-774	-10.75
	T4 → T7	72	0	0.21	-214	-2.97
	T3 → T6	72	0	1.63	220	3.06

Based on the economic analysis (Table 2,and Table 3), the optimal treatment combinations for maximizing economic returns varied by cultivar: Indira and Melodia achieved the highest gross margins ($1,499/ha and $1,533/ha, respectively) and best ROI (89.5% and 91.6%) with Treatment T3 (144 kg N/ha, 120 kg K₂O/ha), while Carma and Mallak performed best with Treatment T2 (144 kg N/ha, 60 kg K₂O/ha), yielding gross margins of $2,018/ha and $1,849/ha and ROIs of 123.4% and 113.1%, respectively. Shantala excelled with Treatment T1 (144 kg N/ha, 0 kg K₂O/ha), achieving a gross margin of $2,650/ha and an ROI of 166%, though T6 (216 kg N/ha, 120 kg K₂O/ha) also performed well. Sensitivity analysis revealed that a 20% increase in fertilizer prices shifted optima toward lower-input treatments, reducing gross margins for high-input treatments by 8–12%, while a 10% decrease in sugar beet prices lowered profitability by 15–22%, though optimal treatment rankings remained stable. Doubling quality premiums favored lower N treatments for Indira, Carma, and Mallak, while Shantala benefited most from T6 due to its high purity. Long-term considerations highlighted potential soil nutrient depletion with low-input treatments, environmental risks from high N applications, and the advantages of moderate inputs given fertilizer price volatility and processing efficiency gains from higher-quality beets. Recommendations included cultivar-specific fertilization (T3 for Indira and Melodia, T2 for Carma and Mallak, and T1 or T6 for Shantala), adjustments based on input costs, quality-focused production for premium markets, and risk management strategies favoring stable returns with Carma (T2) or higher potential returns with Shantala (T6).

## Discussion

The application of nitrogen and potassium fertilizers in sugar beet cultivation has significant implications for environmental sustainability. While these nutrients are essential for optimizing yield and quality, their excessive or improper use can lead to adverse environmental impacts, including nutrient leaching, soil degradation, and water pollution^31,32^. Nitrogen, particularly in the form of nitrate (NO₃⁻), is highly mobile in soil and prone to leaching, especially in regions with high rainfall or excessive irrigation. Leached nitrogen can contaminate groundwater and surface water bodies, leading to eutrophication, which disrupts aquatic ecosystems and compromises water quality^32^. In this study, the use of higher nitrogen levels (288Kg) may increase the risk of leaching, particularly in soils with low water-holding capacity.

The analysis of yield and quality traits uncovered significant differences in the sugar beet cultivars, emphasizing the need for cautious cultivar selection and the opportunity for focused breeding endeavors to enhance specific traits for diverse production environments and market requirements^33–36^. One of the most important findings was the exceptional performance of the Shantala cultivar in terms of root yield (RY), biological yield (BY), Total sugar yield (SY), and recoverable sugar yield (RSY), which indicates that Shantala has a promising ability to efficiently accumulate biomass and allocate resources towards economically valuable traits, such as sugar production. Shantala exhibits high RY and BY values, which can be attributed to its genetic composition. This genetic makeup may provide traits like strong growth, effective nutrient absorption, and resilient photosynthetic machinery, all of which contribute to greater biomass accumulation^25,37,38^.

Our analysis revealed significant cultivar-dependent variations in yield and quality parameters. Shantala demonstrated superior performance in yield traits, recording the highest RY (75.16 t/ha), BY (107.76 t/ha), SY (13.35 t/ha), and RSY (10.58 t/ha). In contrast, Indira recorded the lowest values for these parameters (55.72 t/ha, 91.48 t/ha, 10.07 t/ha, and 7.93 t/ha, respectively). For quality traits, Mallak exhibited the highest sucrose content (18.92%), while Indira showed the highest purity level (79.93%). The alkaline coefficient, an indicator of impurities, was lowest in Carma (6.99%) and highest in Melodia (9.05%). Sugar loss to molasses ranged from 3.24% in Mallak to 4.07% in Melodia. These findings highlight the importance of cultivar selection based on specific production objectives. Shantala would be recommended for high productivity scenarios, while Mallak and Indira would be preferable when sugar quality is the priority.

Moreover, the high SY and RSY values in Shantala show that this cultivar not only produces biomass extremely well but also demonstrates an amazing capacity to direct these resources toward the accumulation and recovery of sugar. Shantala is a viable commercial cultivar in areas where optimizing sugar yield and recovery is a top concern because of this feature combination. Though Shantala showed lower purity and sucrose content (SC) than some other cultivars, its overall yield performance indicates that breeding efforts aimed at enhancing sugar quality characteristics, together with its inherent yield potential, could result in the development of even more productive and efficient cultivars. The observed differences in alkalinity index (AC) and Sugar loss to molasses (SLM) among the sugar beet cultivars highlight the need to take these traits into account in breeding programs and cultivar selection procedures^37,39^. Lower AC and SLM cultivars, including Mallak and Carma, are preferred because they can help to increase the efficiency of sugar extraction and lower processing losses, which raises the total profitability of sugar production. Furthermore, since Total soluble solids (TSS) has been demonstrated to affect sugar output and processing efficiency, the differences in TSS content across the cultivars are particularly important.

This study also captures the significant complex interaction relationships between N and K fertilization treatments, genotypes, and yield and quality parameters in sugar beets. These significant differences have indicated a strong interaction between cultivar and fertilizer treatments, emphasizing the need to tailor nutrient management regimes for every cultivar concerning the desirable traits^37,39^. The results showed that Cultivar Shantala has the highest RY and BY under moderate N and high K (T2) and low N and moderate K (T1) respectively. This means that though beneficial input of nitrogen is essential for root biomass production^40^, higher potassium levels may have a crucial role in improving the over biological yield of this cultivar^1,41^. On the other hand, Melodia cultivar achieved the highest TSS under the T2 treatment, suggesting that moderate nitrogen and high potassium content may have a positive effect on TSS in this cultivar. It was noted that cultivar Indira depicted the maximum SYE under treatment T3 (high N, high K) and Shantala reached maximum SY under treatment T6 (high N, moderate K). These results imply that the regulation of SYE and that of SY may be nutrient-dependent in a cultivar-specific manner^20,25^. Interestingly, nutrient treatments affect sugar content and purity, depending on the cultivar. Mallak plants exhibited the greatest sucrose across treatments. As shown by the genotype’s maximal sucrose content (SC %) under T3 (high N, high K), it might retain more sucrose. The Mallak cultivar, when subjected to the T1 treatment (low N, moderate K), showed the lowest AC and SLM, suggesting that a combination of moderate potassium inputs and limited nitrogen could be beneficial in reducing impurity and sugar loss during processing in this specific genetic background. Furthermore, such variation detected in AC and SLM parameters in response to the interactive effect of both cultivar and treatment which represents the complex genetic-environment interactions, emphasizing the necessity for cultivar-specific optimization methodologies^1,20,22,25,42^. K is a key element for crop growth and productivity^43^. It is an essential nutrient for photosynthesis and the transport of assimilates^44^. K affects the osmotic adjustment of the plant and by enhancing the translocation of assimilates and maintaining osmotic charge^21,45^. Since the transport of Na^+^ from roots to leaves can also be restricted by a high-affinity K^+^ transporter^46^, K supply is a crucial act for crop protection in saline soils.

Principal Component Analysis (PCA) is a robust technique employed in research to discern patterns and correlations among variables in extensive datasets. Scientists have used PCA biplots in sugar beet farming to understand the complex relationships between sugar beet genetic makeup, nutrient management techniques, and the manifestation of important crop yield and quality characteristics. Researchers can analyze the interplay between these elements and see the responses of various cultivars by employing Principal Component Analysis^47,48^. The cultivar-specific responses observed in PCA underscore the importance of tailoring nutrient management strategies to individual sugar beet cultivars. The intricate physiological processes that govern the partitioning and allocation of resources within the plant may account for differences observed between maximizing desirable characteristics such as SC, purity, recoverable sucrose yield, and root yield, and minimizing undesirable characteristics such as sugar loss to molasses and alkaline coefficient. These compromises may result from the complex interplay between yield and quality characteristics, as well as the intrinsic metabolic and genetic limitations of specific cultivars^49–51^.

It is worth noting that cultivars like Indira and Carma showed higher sucrose and RSY levels, while having lower SLM and AC levels, when N and K levels were lower (N1K1 and N1K2). This suggests that excessive N fertilization may have a negative impact on sugar beet quality. When N levels are high, the accumulation of non-sucrose compounds such as inorganic ions and organic acids can occur more rapidly. This can affect the alkaline coefficient and purity of the sucrose. In addition, an overabundance of nitrogen fertilization can encourage the growth of vegetation^52,53^, resulting in a greater ratio of shoots to roots. This can potentially cause a decrease in the allocation of resources towards the accumulation of sucrose in the roots. On the other hand, cultivars like Mallak and Shantala showed better yield and quality parameters such as RY, SY, and SYE when they had higher levels of nitrogen and moderate to high levels of potassium^54,55^. This could be due to the fact that these cultivars have different nutrient requirements and utilization efficiencies. Certain cultivars may require a greater amount of nitrogen and potassium to ensure optimal growth and sucrose accumulation^56–59^, whereas others may be better at utilizing limited nutrient resources. The findings have significant implications for breeding programs and selecting cultivars^47,60^. In addition, the identification of cultivars that respond well to specific nutrient regimes could help in creating more effective nutrient management strategies.

The hierarchical clustering analysis offers valuable insights into the complex connections between nitrogen and potassium fertilization levels, yield traits, and quality parameters among the five sugar beet cultivars^37^. It is worth noting that there is a remarkable association between RY and biological yield BY, as they are tightly clustered together. This finding supports recent studies that have highlighted the significance of biomass accumulation in influencing root yield in sugar beets. The cluster analysis was utilized to categorize 215 sugar beet germplasms into four separate groups, facilitating the identification of eligible groups for the purpose of breeding new kinds, such as energy beet, fodder beet, or sugar beet^37^. In the current study, the distinct cluster formed by TSS, SY, SC, and RSY, reveals moderate to strong positive relationships among these sugar-related traits, which highlights the interdependence of these parameters in determining the overall sugar productivity and quality in sugar beets. The clustering analysis also revealed the co-variation of AC and SLM, forming an interlinked cluster. This finding underscores the importance of these traits in determining sugar extraction efficiency and processing quality, as supported by recent studies investigating the impact of AC and SLM on sugar recovery and processing^22,25^. Interestingly, the distinct cluster formed by purity far from the other traits represents a divergent response, suggesting that the loss to molasses, which is directly related to purity, is regulated by distinct underlying mechanisms compared to the other evaluated characteristics. Recent research has explored the role of various factors, such as impurities, sugar beet storage conditions, and processing techniques, in influencing purity and molasses loss^39,61–63^.

In consistency with results of PCA, the clustering analysis revealed cultivar-specific patterns in response to different nutrient treatment combinations. For cultivars like Indira and Carma, lower nitrogen and potassium levels (N1K1, N1K2, and C1N1K3) clustered favorably with desirable traits like high sucrose, purity, and yields, while higher levels associated with undesirable traits like higher SLM and AC. These findings are consistent with recent studies that have reported the negative impact of excessive nitrogen fertilization on sugar beet quality, leading to increased impurities, reduced SC, and enhanced vegetative growth^1^. Conversely, the higher N and moderate to high K levels (N3K2 and N3K3) for cultivars like Mallak and Shantala, clustered positively with favorable yield and quality traits, such as TSS, BY, RY, and S. However, these treatment combinations also exhibited some affinity towards undesirable traits like higher SLM and AC. These observations align with recent research on cultivar-specific responses to nutrient management strategies, highlighting the need for tailored approaches to optimize yield and quality while minimizing potential trade-offs.

The correlation analysis unveiled noteworthy associations between a multitude of yield and quality characteristics in sugar beet, thereby providing valuable insights into the determinants that contribute to enhanced productivity and quality. The strong positive relationships found between RY and BY, SY, and RSY highlight the vital role of biomass accumulation and partitioning in influencing sugar production and yield in sugar beet and further emphasize the interconnectedness of root development, resource allocation, and the final expression of economically valuable traits such as SY and RSY. These finding are in line with those previously reported^47,48,64,65^. Such strong positive correlations can pinpoint that the sugar yield in sugar beet is influenced by a variety of factors, including both quantitative and qualitative traits. These traits include root yield, sugar content, alpha amino nitrogen, sodium, and potassium^30,66–68^.

Significant cultivar-dependent variations were observed in sugar beet yield and quality parameters, with Shantala demonstrating superior performance in yield traits while Mallak and Indira excelled in quality parameters. The interaction between cultivars and nitrogen/potassium fertilization treatments revealed distinct response patterns, highlighting the inadequacy of blanket fertilization recommendations. Principal Component Analysis identified key relationships between treatments and traits, showing that Indira and Carma performed better with lower fertilizer levels while Mallak and Shantala thrived under higher nitrogen with moderate to high potassium applications. Hierarchical clustering revealed interdependencies between traits, with root yield strongly correlating with biological yield, recoverable sugar yield, and sugar yield. Economic analysis determined cultivar-specific optimal treatment combinations, with Indira and Melodia achieving highest returns with T3 treatment, Carma and Mallak performing best with T2, and Shantala showing exceptional performance with T1, translating to practical recommendations for farmers based on whether they prioritize processing quality, yield maximization, or economic returns.

## Conclusions

Based on the study, nitrogen and potassium fertilization significantly influences sugar beet yield and quality parameters in a cultivar-specific manner, with distinct nutrient response patterns identified across five commercially important cultivars through comprehensive multivariate analyses. The research advances sugar beet agronomy by demonstrating the need for cultivar-specific fertilization strategies rather than uniform recommendations, with Indira and Carma responding optimally to lower nutrient levels while Mallak and Shantala performed better under higher nitrogen with moderate potassium levels. Economic analysis revealed substantial profitability differences, with highest returns achieved by Shantala (T1, 166% ROI), Carma (T2, 123.4% ROI), and Mallak (T2, 113.1% ROI). The study highlights important quality-yield trade-offs that should be considered based on market demands—selecting cultivars like Mallak and Indira with higher sucrose content when processing quality is prioritized or choosing Shantala with treatments T2/T6 when yield is paramount. Optimizing fertilizer application based on cultivar requirements can substantially reduce costs and environmental impacts while providing practical benefits for farmers (40–80% ROI improvements), breeders (development targets), industry (quality enhancement), and policymakers (incentive programs). Although specific to Egyptian conditions, the methodology and principles of cultivar-specific nutrient management have broader application to similar sugar beet growing regions.

## Electronic supplementary material

Below is the link to the electronic supplementary material.

Supplementary Material 1

## Data Availability

“The data that support the findings of this study are available on request from the corresponding author”.
