# Supplementary material for: Cultivar specific nitrogen and potassium recommendations optimize yield and quality attributes in sugar beet
Source: Sci Rep. 2025 Jul 29;15:27550. doi: 10.1038/s41598-025-10918-x (PMC12307683; doi:10.1038/s41598-025-10918-x)

Cultivar−Specific N and K Fertilization for Optimizing Sugar Beet Yield and Quality

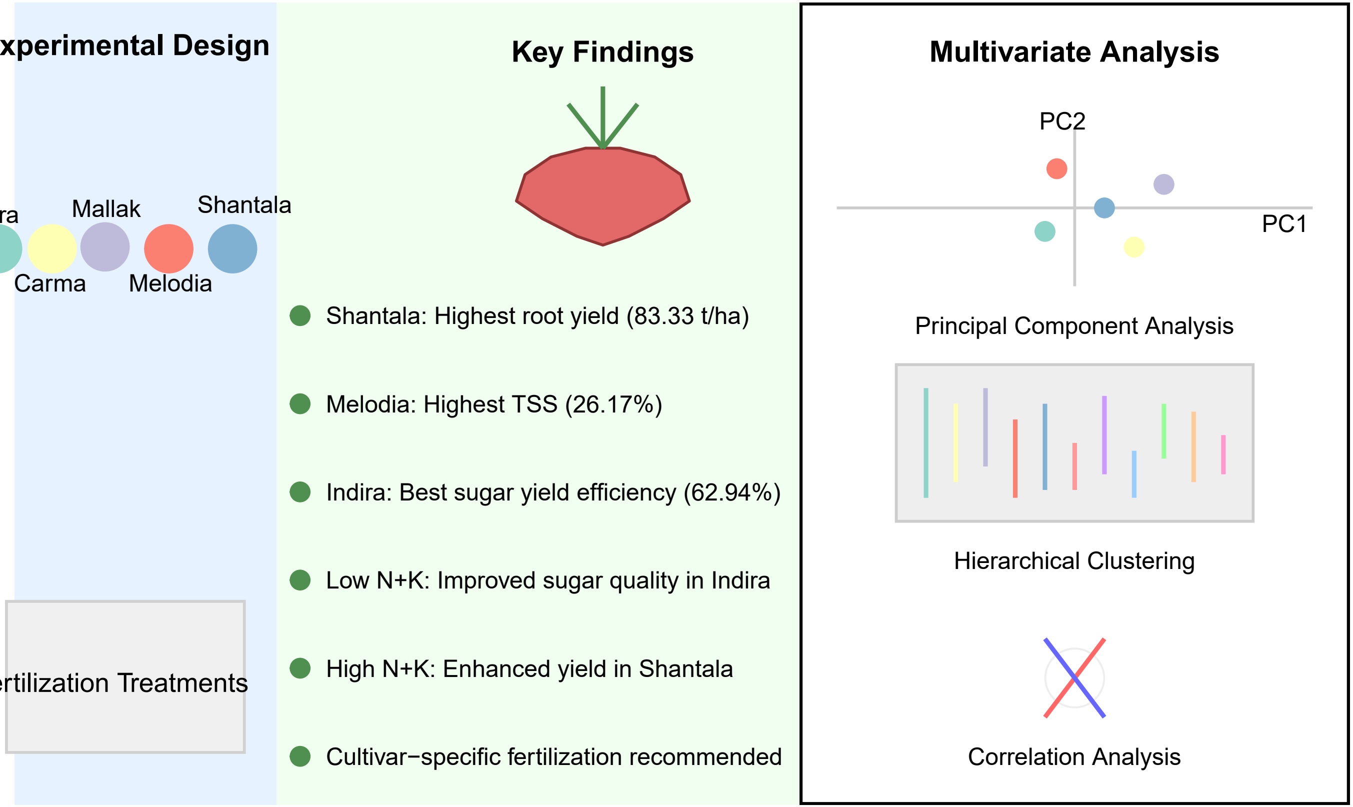

Supplement: Supplementary file 1 — Supplementary Material 1 [file 41598_2025_10918_MOESM1_ESM.pdf]
